# Supplementary figures and images for: TIMP-1 Attenuates the Development of Inflammatory Pain Through MMP-Dependent and Receptor-Mediated Cell Signaling Mechanisms
Source: Front Mol Neurosci. 2019 Sep 20;12:220. doi: 10.3389/fnmol.2019.00220 (PMC6764257; doi:10.3389/fnmol.2019.00220)

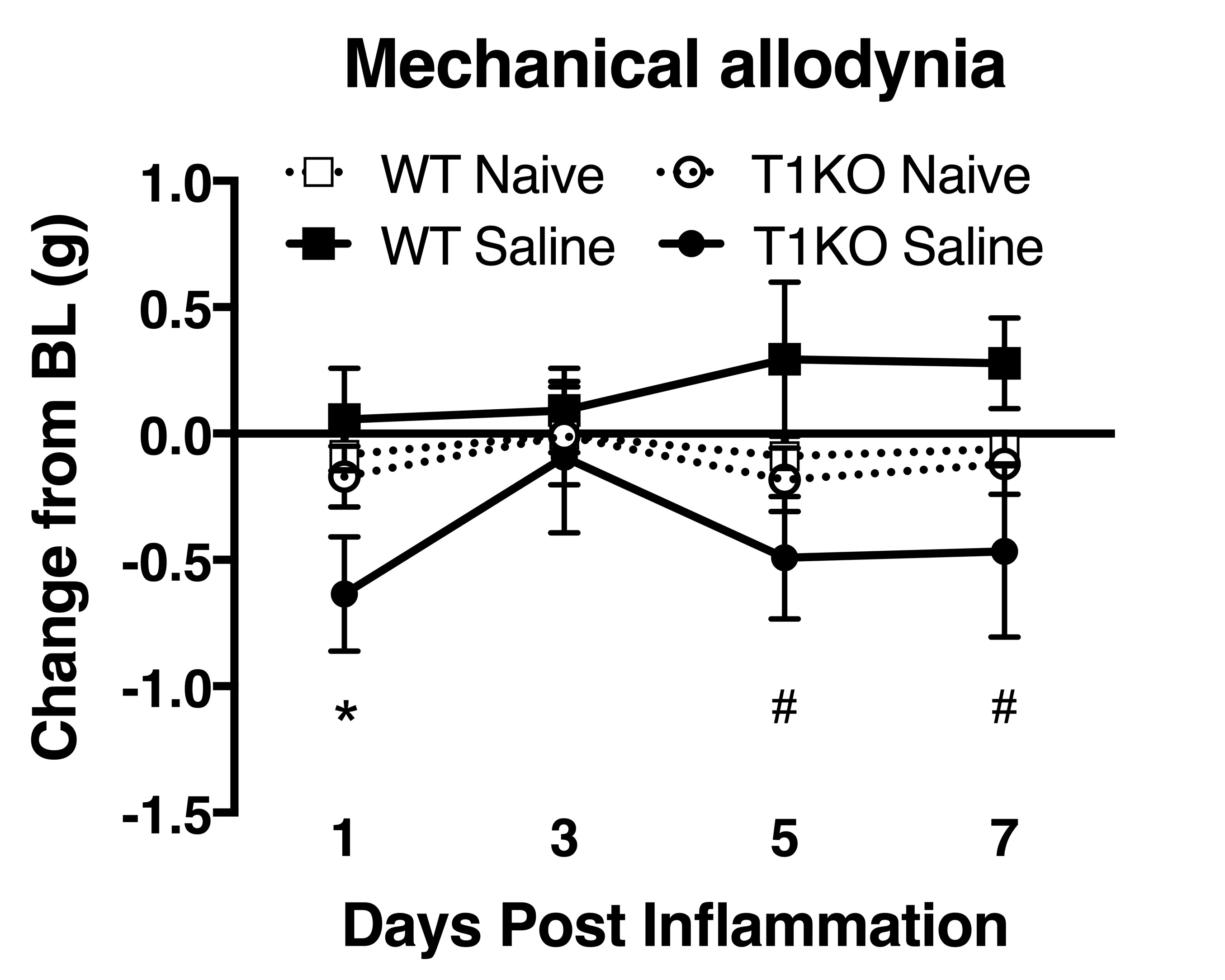

Supplement: FIGURE S1 — Subcutaneous injection of saline vehicle causes mechanical hypersensitivity in mice lacking TIMP-1. Assessment of mechanical hypersensitivity over 7 days following s.c. administration of 0.9% saline. T1KO mice show a significant reduction in mechanical thresholds relative to all other mice 1 day following injection, and saline treated WT mice 5- and 7-days following injection (n = 6/condition). ∗ indicates significant differences compared to naïve controls, and # indicates significant difference compared to WT mice given saline injections, p < 0.05, and error bars depict SEM. [file Image_1.JPEG]

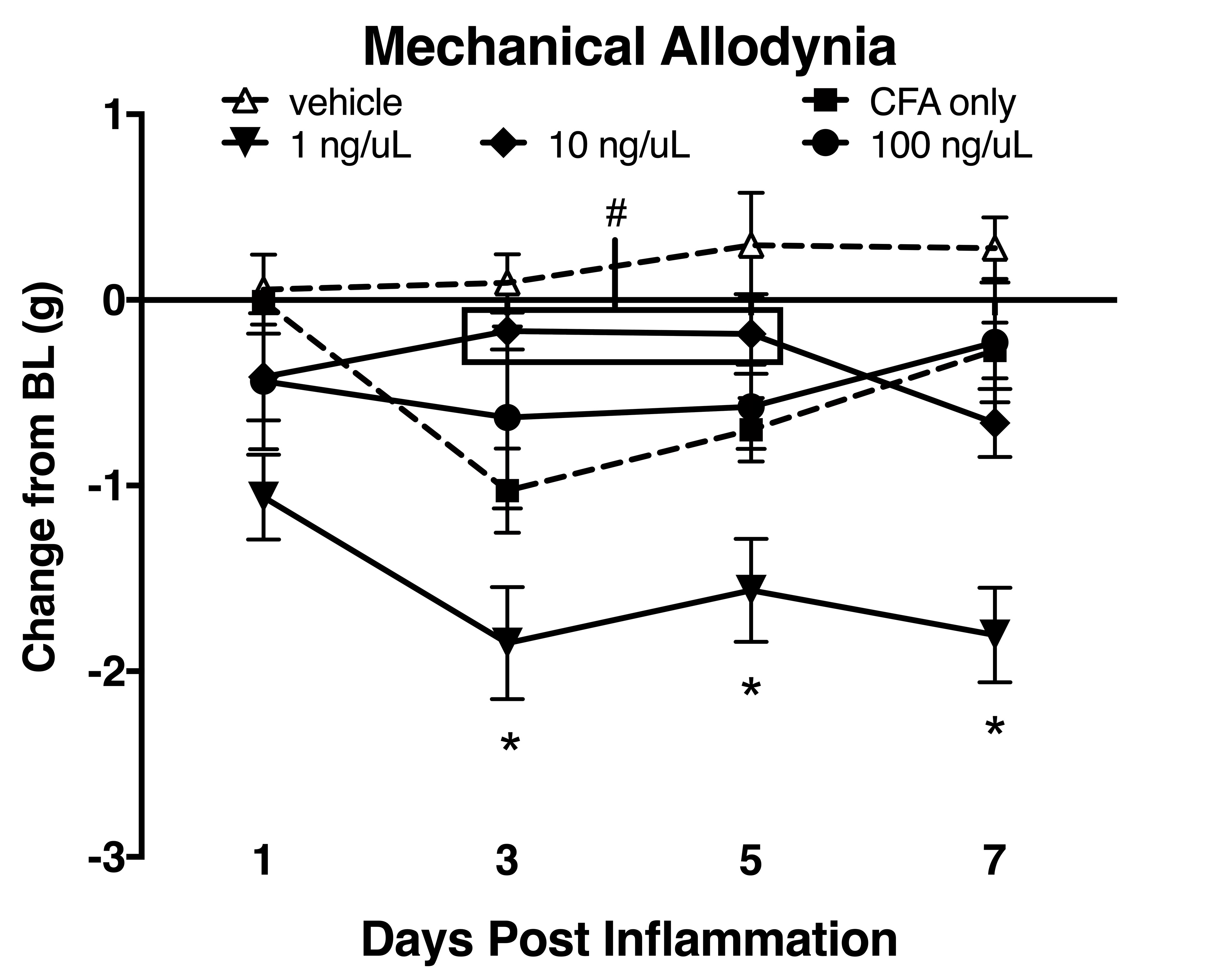

Supplement: FIGURE S2 — Dose-response curve for rmTIMP-1 administration. Mice were administered 1, 10, or 100 ng/μL (10 μL vol) s.c. at the time of CFA injection. Administration of 10 μL of rmTIMP-1 resulted in the greatest attenuation of mechanical hypersensitivity relative to all other doses. ∗indicates significantly different response thresholds relative to all other mice, and #indicates significantly different response thresholds relative to CFA treated mice, n = 6/condition, p < 0.05, and error bars depict SEM. [file Image_2.JPEG]
